# Transcriptional analysis of *Mycobacterium fortuitum *cultures upon hydrogen peroxide treatment using the novel standard *rrn*A-P1

**DOI:** 10.1186/1471-2180-8-100

**Published:** 2008-06-19

**Authors:** María Carmen Núñez, María Carmen Menéndez, María José Rebollo, María J García

**Affiliations:** 1 Departamento de Medicina Preventiva, Facultad de Medicina, Universidad Autónoma, c/Arzobispo Morcillo, 4, 28029-Madrid, Spain; 2 Departamento de Biotecnologia del INIA, Carretera de La Coruña, Km 7.5, 28040-Madrid, Spain; 3 GlaxoSmithKline R&D, Diseases of the Developing World, Molecular Drug Discovery, C/Severo Ochoa, n° 2, 28760-Tres Cantos, Madrid, Spain

## Abstract

**Background:**

The ability of an intracellular pathogen to establish infection depends on the capacity of the organism to survive and replicate inside the host. *Mycobacterium fortuitum *is a bacteria that contains genes involved in the detoxification of the oxygen reactive species such as those produced by the host during the infection. In this work, we investigate the effects of hydrogen peroxide on the transcription and expression of these genes by developing a real time quantitative PCR technique (qRT-PCR) using the ribosomal promoter region (*rrn*A-P1) as reference product for quantification of the mRNA levels.

**Results:**

*M. fortuitum *cultures were treated with different hydrogen peroxide concentrations (0.02 to 20 mM) during several periods of time (30 to 120 minutes). The activity of the enzymes KatGII and SodA, and the transcription of corresponding genes were evaluated. The transcriptional regulator *fur*AII gene was also studied.

The ribosomal promoter region *rrn*A-P1 was validated as referential product under the stress conditions checked by qRT-PCR.

Minor changes were observed under the conditions tested except when bacteria were incubated in the presence of 20 mM hydrogen peroxide. Under those conditions, the levels of transcription of the three genes under study increased at 30 minutes of treatment. The viability of the bacteria was not influenced under the conditions tested.

**Conclusion:**

In this work, we have quantified transcriptional responses to stress suggesting that, the opportunistic pathogen *M. fortuitum *is more resistant and differs in behaviour in the presence of hydrogen peroxide, when compared to the major pathogen *Mycobacterium tuberculosis *and the saprophyte *Mycobacterium smegmatis*. Besides, we demonstrate the mycobacterial non-coding region *rrn*A-P1 to be a suitable reference product in the analysis of qRT-PCR transcriptional data of *M. fortuitum*.

## Background

The genus *Mycobacterium *includes species found in a wide range of ecological niches. *M. tuberculosis*, *M. leprae*, and *M. avium *subsp. *paratuberculosis*, are pathogenic species causing clinical disease in humans or animals. Other species as *M. smegmatis *and *M. phlei *are saprophytes. In addition, an intermediate position is occupied by species opportunistic pathogens such as *M. fortuitum*, a rapidly growing mycobacteria that is ubiquitous in soil and water. This mycobacteria is known to cause cutaneous infection, typically in association with trauma or clinical procedures [1,2]. Due to its capacity to growth and to survive intracellularly, *M. fortuitum *has been previously used as a model for studying the intracellular killing of mycobacteria [3,4].

Most bacteria, when exposed to toxic oxygen metabolites, exhibit an adaptive response and express several genes involved in detoxification of oxygen reactive species, such as superoxide dismutases (SOD), catalases and peroxidases [5,6]. This type of adaptative response has been implicated in the intracellular survival of pathogenic mycobacteria, promoting their maintenance in the host [7-9].

The major pathogen, *M. tuberculosis*, produces an iron co-factored SOD encoded by the gene *sod*A [10] and a copper and zinc SOD encoded by the gene *sod*C [11] whose involvement in protection against oxidative stress of *M. tuberculosis *within macrophages is currently controversial [12,13]. SOD encoded by *sod*A is a major secreted protein [14] that inhibits host responses to *M. tuberculosis *[15]. The expression of genes *sod*A and *sod*C is considered constitutive and inducible respectively [15,16]. Homologues of these genes have been detected in several mycobacterial species, including *M. fortuitum *[13,17].

There are two classes of catalases identified in mycobacteria: the heat-labile catalase or T-catalase (coded by *kat*G), which has also a peroxidase-like function, and the heat-stable catalase or M-catalase (coded by *kat*E). The catalase codified by *kat*G is synthesized preferentially in response to oxidative stress whereas the catalase codified by *kat*E is produced in response to nutrient depletion as occurs in the stationary phase of growth [18]. *M. fortuitum *produces both catalases however *M. tuberculosis *produces only T-catalase whilst *M. terrae *only produces M-catalase [19]. The *kat*G gene has been widely studied in *M. tuberculosis *[20,21]. The KatG protein contributes to the ability of *M. tuberculosis *to grow and survive within the infected host tissues [22] and is involved in the resistance of *M. tuberculosis *to the first-line antimicrobial drug isoniazid [23,24]. Due to their involvement in disease pathogenesis, both *sod*A and *kat*G genes are considered virulence factors of *M. tuberculosis *[25].

The oxidative stress response in bacteria is linked to the iron metabolic pathway [26]. One of the proteins involved in that link is the ferric uptake regulator protein (FurA) an homologue of the Fur of *E. coli*, encoded by the *fur*A gene [27]. The *fur*A gene is located immediately upstream of the *kat*G gene in *M tuberculosis*, an arrangement that appears to be common amongst mycobacterial genomes [28,29].

Only few studies have investigated the regulation of expression of detoxifying genes in mycobacteria. Two promoters have been detected that control *kat*G expression in *M. tuberculosis*, one is located upstream of the *fur*A gene and is co-transcribed with *kat*G [30,31]. Moreover, FurA represses *kat*G gene expression in *M. tuberculosis *and *M. smegmatis *[29] and autoregulates its own expression by binding to the Fur-A box, a 23 bp AT-rich sequence located upstream of *fur*A gene, which is overlapping the -35 and -10 region of the corresponding promoter [32,33]. No studies have yet been published on the regulation of mycobacterial *sod *genes.

The genes *sod*A and *kat*G have been previously studied by our group in *M. fortuitum *[17,34]. We have shown that protein SodA of *M. fortuitum *requires manganese as cofactor [17]. This mycobacteria has two genes encoding for two different enzymes with catalase-peroxidase activities, called *kat*GI and *kat*GII (KatGI and KatGII). KatGII is more related to the single *M. tuberculosis *KatG than KatGI [34]. Upstream of both genes we have also found copies of the *fur*AI and *fur*AII genes respectively [EMBL: Y17061 and Y17062]. The *fur*AII-*kat*GII spacer region spans only 33 nucleotides in the genome of *M. fortuitum*, thus suggesting that the two genes might be co-transcribed.

In this work we have further analyzed the transcriptional activity of *kat*GII, *fur*AII and *sod*A genes in *M. fortuitum *under oxidative stress. Putative promoters have been identified, and real-time quantitative PCR (qRT-PCR) applied to the transcriptional analysis of cultures, which have been exposed to hydrogen peroxide.

qRT-PCR technology has been widely used in the analysis of gene transcriptional activity due to its high efficiency, level of sensitivity and specificity [35-37]. Quantitative gene-expression assays using this technique are typically referenced to an internal control to account for differences in the RNA load. The conditions of the experimental design should not influence the detection level of the selected internal control. Thus the validation of reference controls is recommended in any such experimentation [38].

Two different products *sig*A [39-41] and *rrs *(16S rRNA) [42,43], have most often been used as reference standards in qRT-PCR assays involving mycobacteria. However both of these have disadvantages. *sig*A expression has been found to decrease threefold during entry of bacterial growth into the stationary phase [44]. Levels of *rrs *usually do not change during most phases of bacterial growth, however it is present in a much higher amounts than any mRNA in the cell [M. C. Nuñez & M. J. Garcia, unpublished results] probably because of its higher stability as part of the ribosomes, compared to the stability of other RNA molecules in the cytoplasm, which can cause problems with sensitivity.

Recently, our laboratory described [45] a putative new reference product (*rrn*A-P1) suitable for qRT-PCR. Here we demonstrate the usefulness of this promoter as reference in the qRT-PCR analysis of cultures of *M. fortuitum *during oxidative stress. This methodology may be applied to other quantitative gene-expression analysis of mycobacteria and other bacterial genera in order to evaluate the influence of any experimental treatment on the transcriptional level of gene expression.

## Results

### Enzymatic activity upon oxidative stress of KatG and SodA proteins

**Figure 1:**
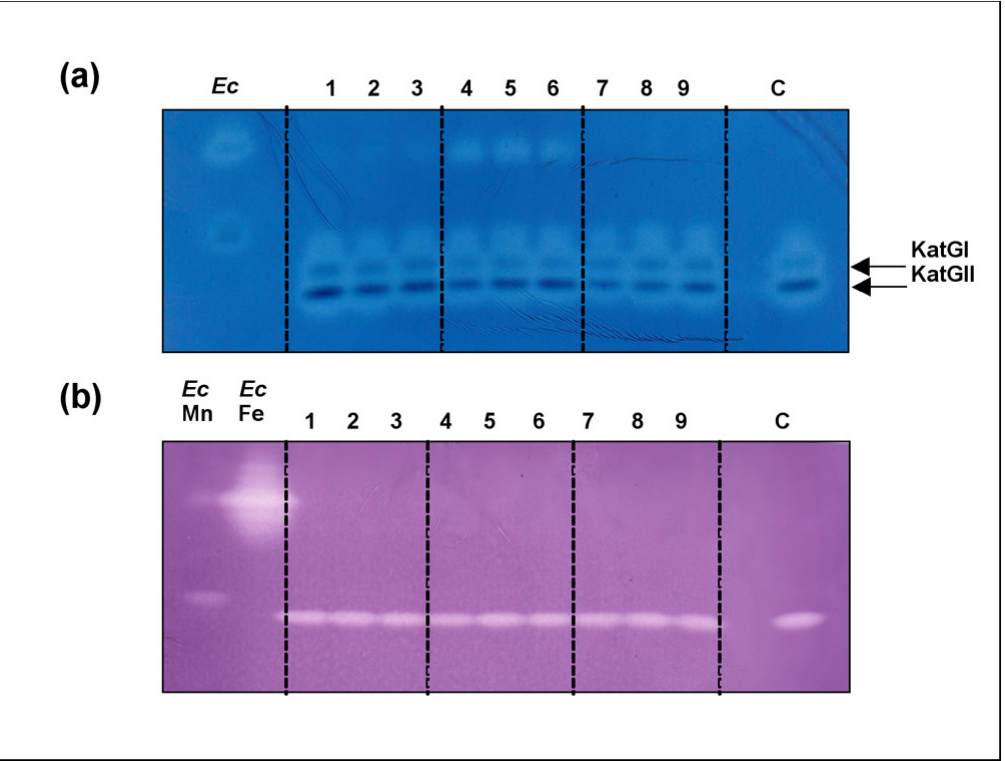
**Protein activity of KatG and SodA using ND-PAGE gels**. (a) Catalase and peroxidase activities; (b) Superoxide dismutase activity. *M. fortuitum *cultures treated with H_2_O_2 _at the following concentrations: Lanes 1 to 3, 0.02 mM; lanes 4 to 6, 2 mM; lanes 7 to 9, 20 mM and lane C, control. Samples of each concentration were treated using increasing length of periods as follows: 30 min (lanes 1, 4, and 7); 60 min (lanes 2, 5, and 8); and 120 min (lanes 3, 6, and 9). The bands corresponding to KatGI and KatGII are indicted with arrows. *E. coli *extracts are incorporated in the catalase-peroxidase activity gel and commercial Fe- and Mn-SOD in the superoxide dismutases activity gel. Abbreviations: Ec: *E. coli*, Mn: Manganese-SOD, Fe: Iron-SOD.

The enzymatic activities of catalase-peroxidase and superoxide dismutase proteins were examined in the extracts collected from *M. fortuitum *cultures (see Methods). The results (Figure 1a) indicated a low level of activity of the KatGI under each of the test conditions. Further analysis of the mRNA levels for *kat*GI (gene coding for KatGI) was unsuccessful, probably due to the scarce expression level of that gene under the experimental conditions applied. The activity of KatGII remains similar from 30 to 120 minutes under 0.02 and 2 mM of H_2_O_2 _(Figure 1a). KatGII showed its lowest activity relative to the control at 30 min of treatment with 20 mM hydrogen peroxide. At this highest concentration, the catalase-peroxidase activity of KatGII increased from cultures treated during 30 min to cultures treated during 120 min (Figure 1a). See Additional file 1 for densitometry values.

The SodA enzymatic activity did not show detectable variation with the control comparing different extracts under all the conditions tested (Figure 1b). This result is in agreement with the considered constitutive expression of the *sod*A gene [15].

### Identification of the transcriptional start points (tsp) of the genes under study

We detected two putative transcriptional start points (tsp P1 and tsp P2) in each the three genes examined by RNA protection assay (RPA) (Table 1a). Putative -35 and -10 boxes were identified according the consensus mycobacterial promoters previously described in the literature [46,47]. The presence of *fur*AII-*kat*GII co-transcripts was tested by standard RT-PCR. Co-transcripts *fur*AII-*kat*GII were detected in all the stress conditions tested (data not shown). These results are in agreement to those described previously in *M. tuberculosis *[30,33].

**Table 1:** Promoter regions of the *sod*A, *kat*GII and *fur*AII genes of *M. fortuitum*

**(a)**								
Gene	P	-35 box	spacing	-10 box	spacing	tsp (+1)	spacing	start codon

*sod*A	P1	TTGTTC	19	TATACG	4	G	95	GTG
	P2	TTGCGG	18	AGTGTT	6	G	44	GTG
*kat*GII	P1	TGCCTG	17	CGGCTT	13	G	90	ATG
	P2	CACGGC	16	CCGAGG	7	T	76	ATG
*fur*AII	P1	TCGTCT	20	CGGAAT	12	T	40	GTG
	P2	TTCTCG	12	TATTCT	5	A	28	GTG

**(b)**								

*Mycobacterium *species	AT-rich region						spacing	start codon

*M. tuberculosis*	AGT CTT GAC TAA TTC CAG AAA AG						9	TTG
*M. smegmatis*	ATT CTT GAC TAA TTC CAG AAA AG						16	GTG
*M. fortuitum *(*fur*AII)	ATTCTT GAC T***A***A TTC CAG AAA AC						16	GTG

(a) Location of the promoters of the *sod*A, *kat*GII and *fur*AII genes of *M. fortuitum*. Abbreviations: P: promoter; -10 box and -35 box: putative -10 and -35 boxes; tsp (+1): transcription start point.

(b) Alignment of the AT-rich sequences upstream of the *fur*A genes in mycobacteria. The -35 boxes of *M. tuberculosis *and *M. smegmatis *[33] and the -10 box of *M. fortuitum *are underlined. The tsp+1 nucleotide of the P2 promoter of the *fur*AII gene of *M. fortuitum *is bolded.

### Validation of *rrn*A-P1 as reference product in qRT-PCR under stress conditions

**Figure 2:**
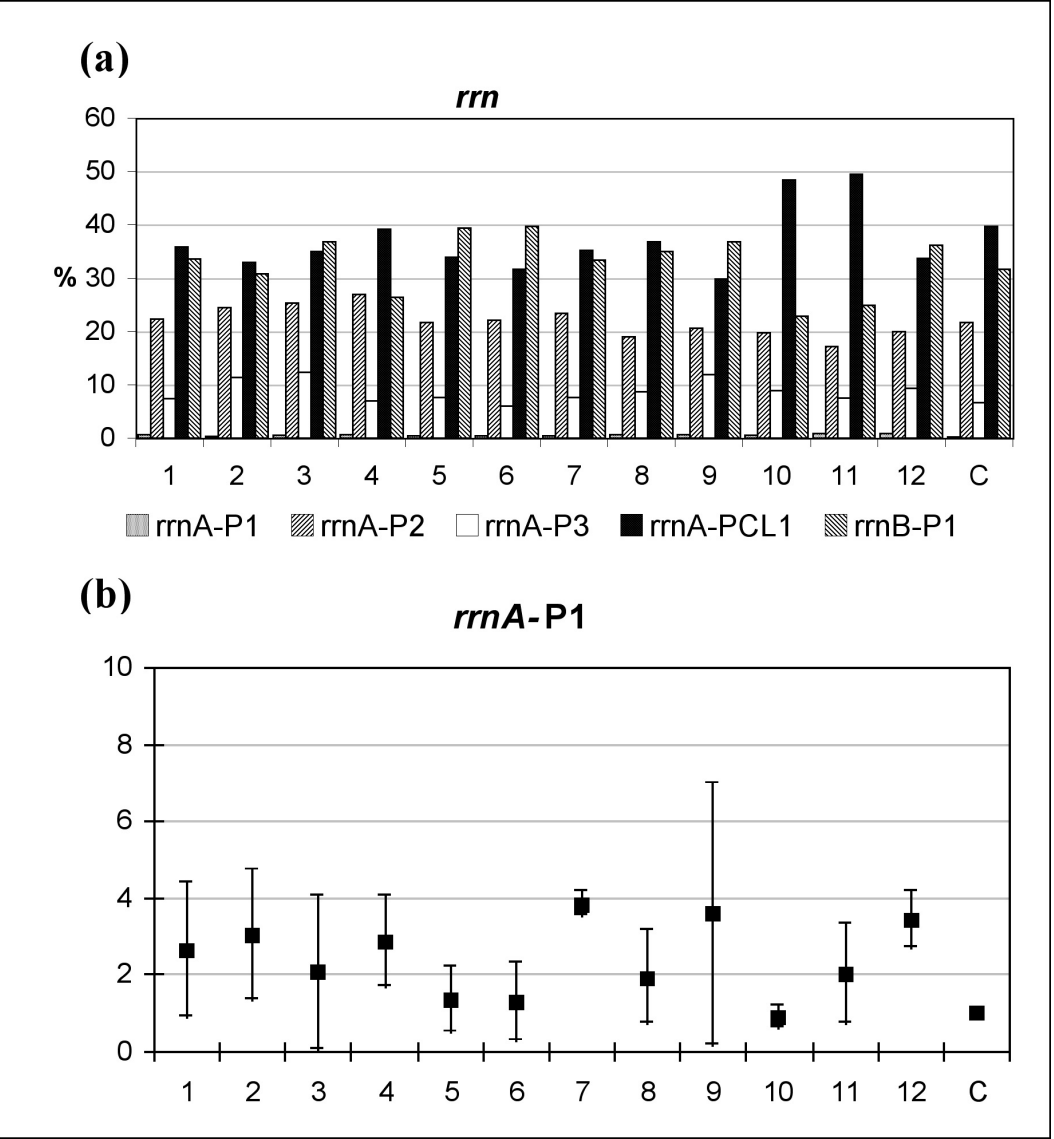
***rrn *promoter-usage under hydrogen-peroxide treatment**. (a) Percentage of usage corresponding to representative experiment of the *M. fortuitum rrn *promoters. *rrn*A-P1 to PCL1 and *rrn*B-P1 indicate the five promoter of *rrn *operons in *M. fortuitum *[48]. (b) Validation of the *rrn*A-P1 promoter as qRT-PCR reference product; by definition, the value of the control is one (see Methods), the statistical significance of data was determined by applying one-way analysis of variance. *M. fortuitum *cultures treated with H_2_O_2 _at the following concentrations: lanes 1 to 3, 0.02 mM; lanes 4 to 6, 0.2 mM; lanes 7 to 9, 2 mM; lanes 10 to 12, 20 mM and lane C, control. Samples of each concentration were treated using increasing length of periods as follows: 30 min (lanes 1, 4, 7 and 10); 60 min (lanes 2, 5, 8 and 11); and 120 min (lanes 3, 6, 9 and 12). Abbreviations: Ct: Cycle threshold.

The *rrn*A-P1 promoter-usage of *M. fortuitum *cultures treated with hydrogen peroxide was analyzed by primer extension (PE) [48,49]. The relative activities of the five *rrn *promoters of *M. fortuitum*, corresponding to different PE products, were determined by quantification of the radioactive levels for each of the conditions tested (Figure 2a).

All the promoters were differentially expressed under each of the conditions tested with the exception of the *rrn*A-P1 promoter whose contribution to the pre-rRNA synthesis was less than 1%, and evenly maintained in each of the stress conditions tested (Figure 2a). These results suggested the suitability of the *rrn*A-P1 promoter as reference standard in qRT-PCR analysis of *M. fortuitum *under oxidative stress.

Experiments were conducted to validate the usefulness of *rrn*A-P1 product as a reference in qRT-PCR, when *M. fortuitum *cultures were grown under oxidative stress. Validation of the *rrn*A-P1 promoter as a constitutive product was investigated using the derivation of the 2^-ΔΔCt ^equation as indicated in Methods: 2^-ΔCt ^(ΔCt = C_t sample _- C_t control_). In this equation the control corresponded to the untreated culture, and the sample corresponded to each stress culture condition. The results are showed in the Figure 2b. No significant differences were found comparing data from all the culture conditions tested with the control (p = 0.445). This result validates the product corresponding to *rrn*A-P1 promoter as a reference in the analysis undertaken.

### Analysis of the *M. fortuitum *gene expression upon oxidative stress

**Figure 3:**
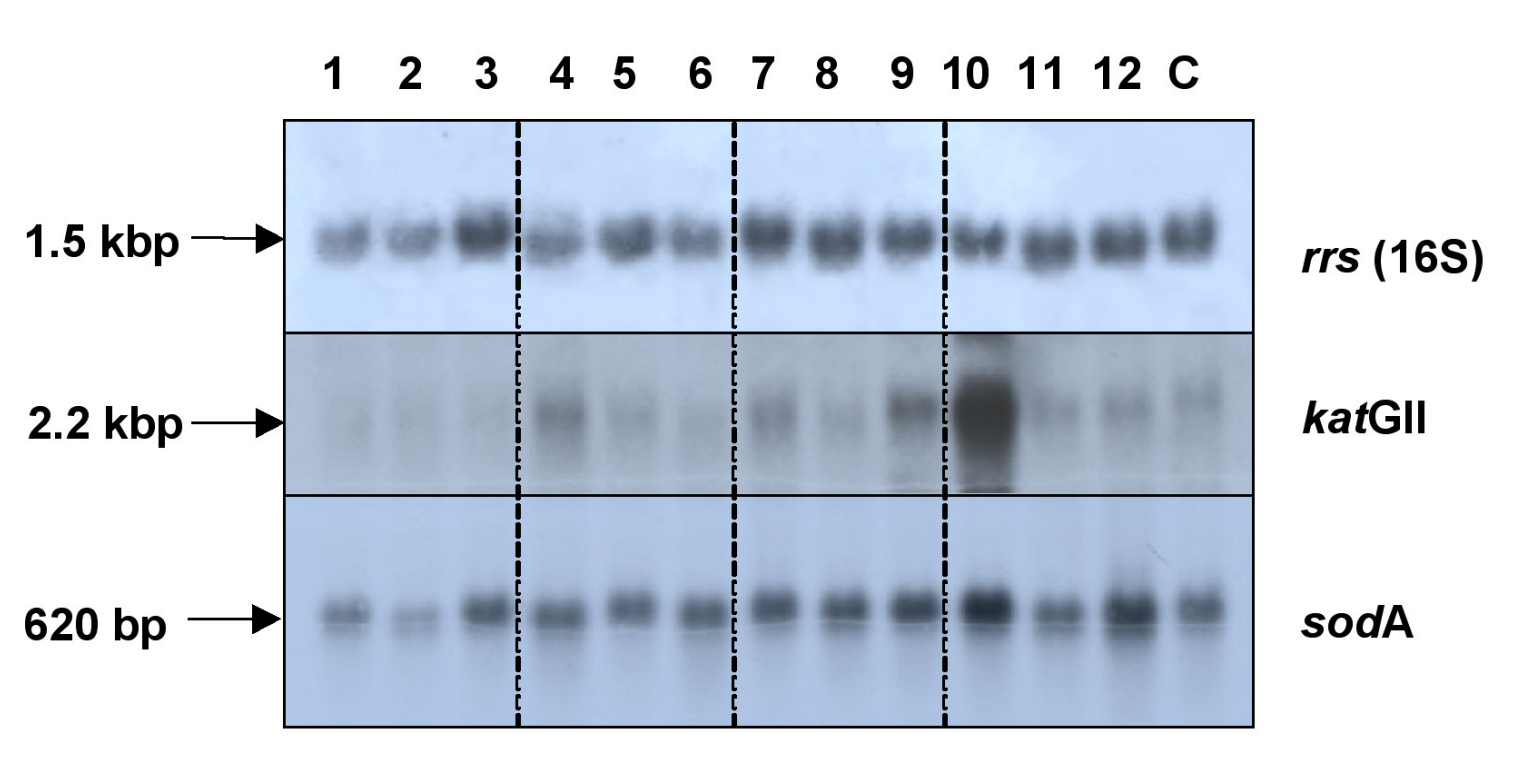
**Northern blot analysis of the *sod*A, *kat*GII and *rrs *under hydrogen-peroxide treatment**. Total RNA from *M. fortuitum *cultures treated with H_2_O_2_: lanes 1 to 3, 0.02 mM; lanes 4 to 6, 0.2 mM; lanes 7 to 9, 2 mM; lanes 10 to 12, 20 mM and lane C, control. Samples of each concentration were treated using increasing length of periods as follows: 30 min (lanes 1, 4, 7 and 10); 60 min (lanes 2, 5, 8, and 11); 120 min (lanes 3, 6, 9 and 12). The same filter was stripped out and re-hybridized sequentially with the several radiolabelled probes: *rrs*, *kat*GII and *sod*A. The sizes of the corresponding mRNAs are indicated.

The transcription of *kat*GII and *sod*A in *M. fortuitum *during the oxidative stress response was initially analyzed using Northern Blot assay (Figure 3). The transcripts were detected by hybridization with internal probes corresponding to each of the genes. An internal probe of the *rrs *gene was also used to test the relative amount of RNA fixed on the filter (Figure 3). A single band was detected per each probe at the expected sizes: 1.5 Kb, 2.2 Kb, and 620 bp for *rrs*, *kat*GII, and *sod*A respectively (Figure 3).

The amounts of radioactive signals corresponding to *kat*GII, and *sod*A genes in each lane were also quantified and normalized with the amount of radioactivity obtained using the *rrs *as a probe (see Additional file 1 for the complete set of the cpm values). No significant changes were obtained in the level of mRNA corresponding to either of the genes under stress conditions, with the exception of mRNA of the *kat*GII gene which increased 10 fold after 30 min of treatment at 20 mM H_2_O_2_; and the mRNA of the *sod*A gene which increased two fold after 30 and 120 min of treatment at the same H_2_O_2 _concentration (Figure 3).

**Figure 4:**
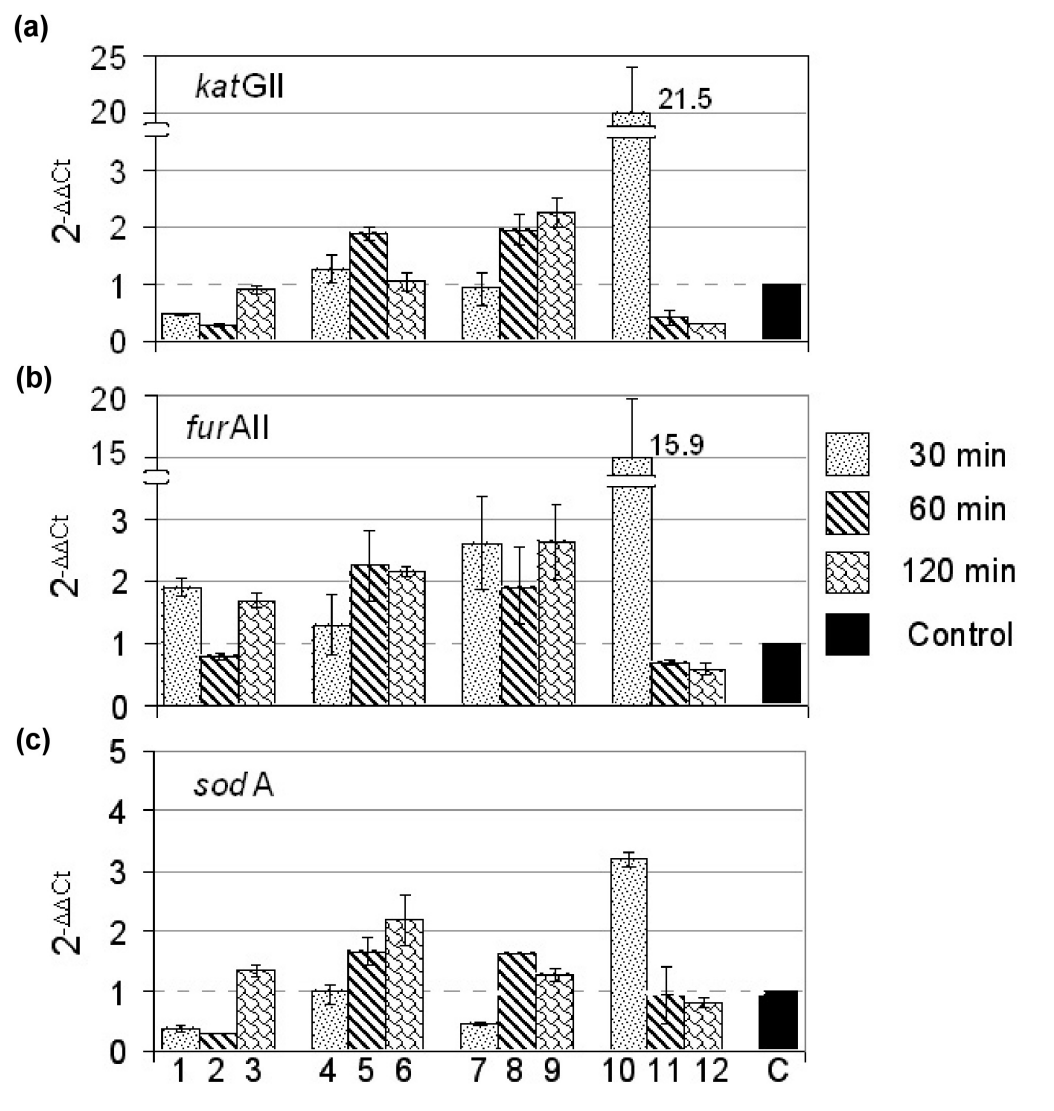
**qRT-PCR analysis of *kat*GII, *fur*AII and *sod*A under hydrogen-peroxide treatment**. (a) *kat*GII gene expression; (b) *fur*AII gene expression; (c) *sod*A gene expression. The 2^-ΔΔCt ^method was applied to calculate the relative amount of the cDNAs of interest, comparing treated and untreated cultures (see Methods). *M. fortuitum *cultures were treated with hydrogen-peroxide at the following concentrations: lanes 1 to 3, 0.02 mM; lanes 4 to 6, 0.2 mM; lanes 7 to 9, 2 mM; lanes 10 to 12, 20 mM and lane C, control. Samples of each concentration were treated using increasing length of periods as follows: 30 min (lanes 1, 4, 7 and 10); 60 min (lanes 2, 5, 8 and 11); and 120 min (lanes 3, 6, 9 and 12).

In order to determine more accurately the amount of the transcripts of *M. fortuitum *in response to oxidative stress, qRT-PCR technique was applied to quantify the mRNAs. The transcription of *sod*A, *kat*GII and its putative regulator *fur*AII were analyzed (Figure 4). The genes under study did not show main variations in the quantity of products in relation to the different H_2_O_2 _concentrations applied, as well as in relation to the duration of the treatment. Again, the only significant change was detected at the highest concentration tested (20 mM H_2_O_2_). At this concentration, the highest level of *kat*GII, *fur*AII and *sod*A expressions corresponded to the culture treated with H_2_O_2 _during 30 min. Compared to the control culture, *kat*GII and *fur*AII mRNA levels product increased in this point more than twenty and fifteen fold respectively. The products corresponding to *sod*A suffered a three-fold increase in this same point of analysis (Figure 4).

Under other H_2_O_2 _concentrations tested (0.02 mM, 0.2 mM and 2 mM) the mRNA levels of *kat*GII, *fur*AII and *sod*A either increased to a lesser extent or remain unchanged with the duration of the treatment (Figure 4).

### Viability of *M. fortuitum *under different H_2_O_2 _stress treatment

**Figure 5:**
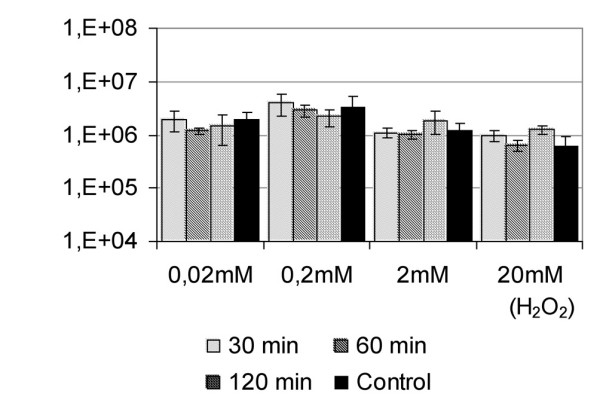
**Viability of the *M. fortuitum *cultures treated with H_2_O_2_**. The colony forming units (CFUs) were determined for the untreated control and for each culture treated with 0.02, 0.2, 2 and 20 mM of H_2_O_2 _after 30, 60 and 120 minutes. One control was used for each H_2_O_2 _concentration (showed as black columns in the figure). The error bars corresponding to data from three different cultures are showed.

To analyse the influence of the stress on the viability of the *M. fortuitum*, we determined the colony forming units (CFUs) of each of the cultures (Figure 5). No main differences were found when controls and test cultures were compared, indicating that changes detected at the mRNA and proteins levels were not related to changes in the bacterial viability.

## Discussion

Mycobacteria are ubiquitous bacteria and are therefore exposed to a wide range of environmental conditions, including animals and humans. One of these conditions is the host stress response which includes induction of reactive oxygen intermediates (ROI) against which mycobacteria synthesize enzymes such as superoxide dismutases, catalases and peroxidases. This work studies these enzymatic responses in the opportunistic pathogen *M. fortuitum *growing in the presence of hydrogen peroxide measured using real-time PCR to quantify mRNA specific products corresponding to genes *sod*A, *kat*GII and *fur*AII involved in the oxidative stress response.

The development of a reliable qRT-PCR method requires corrections for experimental sample variations, such as reverse transcription efficiency. Those variations are best controlled by the inclusion of an internal reference standard. Ideally this reference standard should have a constant expression under each of the experimental conditions [50]. Moreover, validation of the internal control is recommended when designing quantitative gene expression studies [50].

We have previously reported that the first promoter of the *rrn*A operon (*rrn*A-P1) gave better results in *M. fortuitum *[45] than *sig*A and 16S rRNA, the standards more frequently used in *M. tuberculosis *[40,43]. This study has tested this product for suitability as reference standard in the study of the oxidative stress response of *M. fortuitum*.

The primer extension results showed that the product of the *rrn*A-P1 promoter was synthesized at a low and constant level when *M. fortuitum *cultures were placed under oxidative stress (Figure 2a). Experiments conducted to validate *rrn*A-P1 as reference standard in qRT-PCR (Figure 2b) found no statistically significant changes (p = 0.445), in expression levels during several of the experimental conditions. It was concluded therefore that *rrn*A-P1 is a suitable reference standard for qRT-PCR studies of *M. fortuitum *growing under oxidative stress.

In contrast to the single promoter previously detected upstream of *fur*A and *kat*G genes of *M. tuberculosis *[30,31], two promoters were identified in the *M. fortuitum *genome upstream of *fur*AII, *kat*GII and *sodA *(Table 1a). *fur*AII and *kat*GII genes were co-transcribed in *M. fortuitum *in a similar manner to *M. tuberculosis *[30,33], showing the previously described conserved organization of those genes among mycobacteria [29]. Recently, it has been demonstrated in *M. smegmatis *that the single transcript containing *fur*A and *kat*G is processed to originate two different transcripts, one covering *fur*A and the second covering *kat*G [51], that result explain why *fur*A-*kat*G co-transcripts were not detected in this species previously [32]. Nevertheless the occurrence of a similar phenomenon in *M. fortuitum *cannot be excluded, differently to *M. smegmatis, fur*AII-*kat*GII co-transcripts were detected in *M. fortuitum *supporting that a different transcriptional organization could occurs in this last mycobacteria.

Several genomes of mycobacterial species have been sequenced or are in progress [52], including that of the rapidly growing mycobacteria *M. smegmatis *(strain mc^2^155). This strain is used frequently in the study of mycobacteria, because of its rapid growth, and low biosafety requirements. A search of the *M. smegmatis *genomeidentified three putative *kat*G genes. Two of these are associated with a corresponding *fur*A gene immediately upstream, including a Fur-A box in one of them. Also of note is that the third *kat*G gene has a long deletion in its 5' end (190 nucleotides), which could explain the lack of the sequence corresponding to a paired *fur*A gene upstream.

Studies in *M. tuberculosis *have clearly established that Fur-A is a negative regulator of the *kat*G expression in mycobacteria [29,33]; this regulation is mediated by the FurA-box identified upstream of the *fur*A gene in the region corresponding to the -35 promoter sequence, also known as the AT-rich sequence. The linkage of FurA to the FurA-box is inhibited in the presence of hydrogen peroxide thus allowing the transcription of the downstream genes. Sala et al. [33] have analyzed the FurA box of *M. smegmatis*, which presumably included the single *fur*A-*kat*G copy associated with this regulatory sequence. In this work we identify that AT-rich sequence 16 nucleotides upstream the *fur*AII start codon of *M. fortuitum *(Table 1b). This sequence includes the -10 box corresponding to the P2 promoter identified (Table 1). Similarly to *M. smegmatis*, only one of the two *fur*A-*kat*G tandem genes of *M. fortuitum *has a sequence equivalent to the FurA box upstream.

By using qRT-PCR, we observed that the influence of hydrogen peroxide in the level of expression of *kat*GII and *fur*AII of *M. fortuitum *differs from other mycobacteria [29,31,33,53] by not showing variation in the protein and mRNA levels of these genes when *M. fortuitum *was treated with up to 2 mM hydrogen peroxide (Figures 1, 3 and 4). These results could indicate that *M. fortuitum *appears to be more resistant to the hydrogen peroxide stress than other mycobacterial species.

We did find significant changes however in the stress response in cultures of *M. fortuitum *when treated with 20 mM H_2_O_2_. Under such conditions, the mRNA levels of the two genes (*kat*GII and *fur*AII) increased significantly, and the level of *sod*A mRNA rose to its higher level after 30 minutes of treatment (Figure 4). Considering also that only a low level of mRNA could be detected when cultures were treated with 20 mM of H_2_O_2 _during 60 and 120 minutes (Figure 4), we hypothesized that 20 mM H_2_O_2 _may be deleterious for the bacteria, however *M. fortuitum *cultures studied under stress found no decrease in CFU under each of the conditions tested (Figure 5). We conclude therefore that the changes detected in genes related to the stress response (Figure 4) are not associated to changes in the viability of the bacteria, however they could indicate a compensatory reaction, at the transcriptional level, to the decrease detected at the protein level (Figure 1) indicating some adaptative response of the transcriptional machinery to that extremely adverse environmental conditions. Further studies are required to determine if the response to 20 mM H_2_O_2 _might indicate a global alteration in the transcriptional activity or possibly post-transcriptional regulation of *M. fortuitum*.

## Conclusion

Our results indicate that mycobacteria differ in their response against oxidative stress. The opportunistic pathogen *M. fortuitum *is more resistant to hydrogen peroxide than the main pathogen *M. tuberculosis *and the saprophyte *M. smegmatis*. Even though changes in the mRNA and proteins related to oxidative stress were detected, the *M. fortuitum *viability was not affected at a H_2_O_2 _treatment as strong as 20 mM during 120 minutes.

This work also demonstrated the suitability of the non-coding region (pre-*rrn*A-P1) as reference standard in the quantification of the transcriptional activity of genes associated to oxidative stress in *M. fortuitum*. The reference product applied (pre-*rrn*A-P1) is related to the ribosomal RNA, a bacterial component frequently used as reference in the analysis of the bacterial transcriptional activity.

## Methods

### Bacterial strain and growth conditions

*M. fortuitum *ATCC 6841^T ^was maintained on Lowenstein Jensen at 4°C for short-term storage and 40% glycerol at -70°C for long-term storage.

Three different cultures of *M. fortuitum *were grown at 37°C until exponential phase (OD = 0.6–0.8 U) in 100 ml of Sauton broth [54]. Hydrogen peroxide (H_2_O_2_) was then added to obtain 0.02 to 20 mM final concentrations. Cells were further incubated at 37°C with shaking for 1/2 h, 1 h and 2 h, per each H_2_O_2 _concentration. Total RNA and proteins were purified from the cultures (75 ml and 25 ml respectively) following to the treatment with hydrogen peroxide. Untreated cultures were also tested as reference.

Viability of bacteria recovered from the cultures was checked, by determining the colony forming units (CFUs) on Middlebrook 7H10 petri dishes. To reduce possible variation introduced by individual inocula over the several sets of cultures, a separate untreated control culture was included for each H_2_O_2 _concentration tested.

### Protein isolation and analysis of the protein activity

The protein extracts were prepared by vigorous mixing of bacterial cells in the presence of 0.15–0.212 mm of diameter glass beads (SIGMA) during three sequential pulses of 45 s at 4 m/s in a Fast-Prep (Savant-Instruments). Bacterial extracts were centrifuged and the supernatants were recovered and stored at -70°C. The analysis of the extracts was made by Polyacrilamide Gel Electrophoresis (PAGE). Activity gels were performed in non-denaturing polyacrylamide gel electrophoresis (ND-PAGE) [55], 10% and 15% [wt/vol] ND-PAGE were applied for catalase and peroxidase and superoxide dismutase analysis respectively. Catalase and peroxidase activities of the mycobacterial extracts were examined by the double-staining method as described previously [56]. Superoxide dismutase activity was examined as described previously [57,58]. Densitometry of the activity bands was determined directly on the gels using ID-Manager (TDI) for comparative purposes.

### RNA isolation

Mycobacterial cultures were collected, and total RNA isolated as described previously [59]. Quality of the RNA isolation was checked by gel electrophoresis followed by quantification in spectrophotometer [55].

### Analysis of precursors rRNA (pre-rrn) by primer extension

The ribosomal promoter usage of *M. fortuitum *in Sauton cultures, treated and untreated with H_2_O_2_, was determined using the primer extension (PE) procedure. The primer JY15 (Table 2) was end labelled with [γ-32P]ATP by T4 polynucleotide kinase, and primer extension was carried out with Avian Myeloblastosis Virus (AMV) reverse transcriptase (Promega) as described previously [59]. PE experiments were performed with 25 μg of total RNA per sample. Transcriptional products, corresponding to each *rrn *promoter, were quantified in the gels by using an Instant Imager system (Packard-Izasa).

**Table 2:** Oligonucleotides used in this study

**DNA target**	**Forward Primer [Sequence 5'→3']**	**Reverse primer [Sequence 5'→3']**	**Annealing T (°C)**	**Size amplicon (bp)**
***Northen Blot (probes)***				
*rrs*	kk4 [TTGGAGAGTTTGATCCTGGCTCAG]	Rac6 [GTGGACTACCAGGGTATCTAATCCT]	58	791
*sod*A	Sod3.1 [ACATCTCGGGTCAGATCAACGAG C]	Sod4 [GACGTTCTTGTACTGCAGGTAG]	58	465
*kat*GII	Cat39 [AAACCCATCCTACTATCG]	Cat40 [GGTTCACGTAGATCAGG]	50	696
***qRT-PCR***				
*rrn*A-P1	FoP1 [CTGCTCGTCAGCCTCGAAATCG]	P1R-LC [ACTTCAAAAGATTAGCGCGG]	55	75
*sod*A	Sod12 [CCTTCCAGCTCTACGACCAG]	Sod13 [CCTTCCAGCTCTACGACCAG]	55	114
*kat*GII	katLC1 [GTGGTCCGATCTCATCGTCT]	katLC2 [CAGTAGATGTCCTCCTCGGG]	55	117
*fur*AII	furA-Fw [ACGGCGGACTTCCAGCAG]	furA-Rev [ACGGATGATCGTTTCGGTG]	62	120
***RPA***				
*sod*A	Sod F8 [ACCTGCACACCCGCAACC]	Sod 3T [PT7-GCTCGTTGATCTGCCCCGA]	60	324
*kat*GII	Cat35 [GACAACCACCACCACGT]	P1.1 T [PT7-TCTCGGCGGGTTCAGTCT]	50	292
*fur*AII	FUR-II-10 [TCGTCTAGCGCCCTCACC]	FUR-II-1T [PT7-ACTCGGGGTCGGGTCACA]	60	177
***Other primers***				
*fu*rAII-*kat*GII	furAII-Fo [GACCTACCCGAGGTCTCTCA]	P1.1 [TCTCGGCGGGTTCAGTCT]	54	388
pre-*rrn*A	FoPCL1 [CAAAACAGGGCGGCAAAGC]	cKK4 [GAGCCAGGATCAAACTCTCC]	58	225
16S-*rrn*A		JY15 [CACACTATTGAGTTCTC]	-	-

Oligonucleotides used for Norther Blot, RNAse protection assay, real-time qRT-PCR and conventional PCR.

Abbreviations: PT7: RNA polymerase promoter sequence for the phage T7 (AATTCTAATACGACTCACTATAGGG).

### Identification of transcription starts points using RNAse Protection Assay

RNase protection assay (RPA) was carried out as described previously [60]. The RPA method requires previous preparation of a radiolabeled RNA probe complementary to the RNA transcript by transcription of a minigene corresponding to the region under analysis [61]. Minigenes were synthesized by PCR amplification of the *M. fortuitum *corresponding genomic regions by using oligonucleotides described in Table 2.

Radiolabelled transcription products were obtained by using the Riboprobe kit (Promega) as specified by the manufacturer; this yielded probes of 324, 292 and 177 nucleotides for *sod*A, *kat*GII and *fur*AII genes respectively. The products were purified on a polyacrylamide gel electrophoresis (6% [wt/vol] polyacrylamide) sequencing gel. The appropriate bands were excised and eluted as described previously [60].

Hybridization was carried out in PES buffer [60], with radiolabeled samples (2.5 × 10^5 ^cpm) and 20 μg of DNA-free RNA; the reaction products were digested with different amounts of an RNase cocktail (Ambion). Stock RNase cocktail comprises both RNase T1 (20 U/ul) and RNase A (1 μg/μl), and an optimum concentration (up to a 10-fold dilution) was established for use in the RNA protection assays.

Products of both primer extension and RNase protection assays were analyzed by electrophoresis through 6% (wt/vol) polyacrylamide-8 M urea sequencing gels. Radioactive products were located by autoradiography either at 20°C or at -70°C using an intensifying screen. The gels were calibrated with the products of appropriate DNA-sequencing reactions. DNA sequences were determined by the dideoxy chain termination procedure with [α-35S]dATP [55]. The putative promoter regions (-10 and -35 boxes) were determined (Table 1).

### Analysis of *fur*AII-*kat*GII region

The cDNA from *M. fortuitum *under the different stresses was analysed by PCR using the primers furAII-Fo and P1.1 (Table 2) to test the presence of *fur*AII and *kat*GII cotranscripts. PCR cycling was performed as follows: a denaturizing step at 95°C for 5 minutes; 36 cycles of 95°C for 45 seconds, 54°C for 45 seconds and 72°C for 1 minute; and a final extension at 72°C for 10 minutes.

### Analysis of mRNA by Northern blot

Northern blot analysis was performed by fractionation of 20 to 30 μg of total RNA samples on a 1.5% agarose gel containing 6.6% formaldehyde. RNA ladder (0.24 to 9.5 kb; Gibco BRL) was used as the molecular size standard. The gels were transferred to a nylon membrane (Hybond-N; Amersham) by capillary blotting in 20× SSC (1× SSC is 0.15 M NaCl plus 0.015 M sodium citrate), and further cross-linked by UV irradiation [55].

The membranes were hybridized with three probes: *kat*GII, *sod*A and the 16S rRNA (*rrs*) genes sequentially. The probes were prepared by standard PCR. Amplifications were performed on *M. fortuitum *chromosomal DNA (100 ng) in a final volume of 50 μl containing 1 μM of each primer and 1 U of *Taq *polymerase (Amplitaq Gold, Roche). All amplifications were carried out as follows: 36 cycles of 1 min at 95°C; 45 s at the annealing temperature (Table 2); and 1 min at 72°C, with subsequent 10 min final extension at 72°C. The primers used amplify the coding regions of the corresponding gene. Primers characteristics, annealing temperatures, and sizes of each amplicon are indicated in the Table 2. Hibridizations were made with 90 ng of each PCR product labeled with ^32^P α-dCTP using Megaprime DNA labeling system (Amersham) at 65°C overnight. Standard procedures were used for both hybridization and washes [55]. Filters were spread out and rehybridized with the three probes sequentially. The radioactivity hybridized for each gene was quantified on the filter using an Instant Imager system (Packard-Izasa) and also detected by exposition of the membranes to X-ray films. Data derived from hybridization with the *rrs *gene were used as standard to normalize the amounts of RNA fixed on the filter.

### Analysis of mRNA by qRT-PCR

One hundred ng of the mycobacterial RNA isolated was reverse transcribed by using 30 U AMV reverse transcriptase and random primer hexamers (Promega). The absence of DNA following DNase treatment was checked by performing conventional PCR using FoPCL1 and cKK4 (Table 2). Conventional PCR cycling was performed as follows: a denaturizing step at 95°C for 5 minutes; 36 cycles of 95°C for 1 minute, 58°C for 45 seconds and 72°C for 2 minutes; and a final extension at 72°C for 10 minutes. The RNA product was considered suitable for reverse transcription when the control-DNA PCR was negative.

The qRT-PCR method was used to determine the level of each transcriptional-product derived from *M. fortuitum *under the different stresses tested. The qRT-PCR was performed with cDNAs prepared from 2 separate cultures per treatment. A total of 16 data were obtained per point derived from two cDNA separate preparations from each culture. Each of the four cDNA samples obtained was amplified twice in duplicated experiments.

Real-time PCR was carried out using a capillary PCR instrument (Light Cycler; Roche). The amplification of the target sequence was detected using SYBR green Conditions were used as follows: LightCycler Fast Start DNA master SYBR Green I reagent (1 μl) was supplemented with 3.5 mM (final concentration) MgCl_2 _and 0.5 μM of each primer in 7 μl of volume. Sample cDNA (3 μl) was added to the mix. The PCR cycling programme was as follows: denaturizing, 1 cycle of 95°C for 10 min with a transition rate of 20°C/s; amplification, 45 cycles at 95°C for 0 s, the corresponding annealing temperature for each product (Table 2) for 5 s and an extension at 72°C for 10 s with a single fluorescence acquisition, in all the cases the transition rate was 20°C/s. Specificity of the reaction was checked by analysis of the melting curve of the final amplified product. The oligonucleotides used in qRT-PCR are indicated in the Table 2. In all cases, the negative control was undetectable, because the PCR experiments were stopped before its fluorescence increased.

### qRT-PCR data analysis

The qRT-PCR data were plotted as the fluorescence signal versus the cycle number. An arbitrary threshold was set at the midpoint of the log of fluorescent level versus cycle number plot. The Ct value is defined as the cycle number at which the fluorescent level crosses this threshold. In the present study, the fold change in the cDNA amounts was calculated from the formula 2^-ΔΔCt ^(Applied Biosystem User Bulletin). Using the 2^-ΔΔCt ^method, the data are presented as the fold change in gene expression normalized to an endogenous reference gene and relative to the untreated control. This calculation transforms the logarithmic Ct data to a linear value. Error of the data was calculated by converting the data from logarithmic to linear using the expression 2^-Ct^. Raw C_t _values falsely represents the error and should be avoided [50]. For the untreated control sample, ΔΔC_t _equals zero, therefore by definition, the fold change in gene expression relative to the untreated control is one. For the treated samples, 2^-ΔΔCt ^indicates the fold change in gene expression relative to the control [38]. The fold change in cDNA (per each target gene) relative to the *rrn*A-P1 control [45] was determined as follows: fold change = 2^-ΔΔCt^; where ΔΔCt = (Ct_Target _- Ct_*rrn*A-P1_)_Sample _- (Ct_Target _- Ct_*rrn*A-P1_)_Control_. The sample corresponds to each H_2_O_2 _treated cultures, and controls are the untreated cultures in the formula. According to Winer [62] the relative quantification of gene expression by using the 2^-ΔΔCt ^method correlates with the absolute gene quantification obtained using standard curves. The statistical significance of data was determined by applying one-way analysis of variance.

## Authors' contributions

MdCN carried out the molecular biology and protein studies, and participated in the drafted the manuscript. MdCM participated and supervised the design of the study and helped to the elaboration of the manuscript. MJR carried out the identification of tsp of the genes in this study. MJG conceived the study, and participated in its design and follow up and coordinate the manuscript. All authors read and approved the final version of the manuscript.

## Supplementary Material

Additional file 1 Quantitative data corresponding to Figures 1 and 3. Data corresponding to Figure 1 show the densitometry of a representative activity gel of KatG and SOD in protein extracts of *Mycobacterium fortuitum*. Data corresponding to Figure 3 show the cpm values of a representative experiment of hybridization of a Northern Blot with *Kat*GII, *sod*A and *rrs *(16SrRNA) before and after normalization. Click here for file

## References

[B1] Winthrop KL, Albridge K, South D, Albrecht P, Abrams M, Samuel MC, Leonard W, Wagner J, Vugia DJ (2004). The Clinical Management and Outcome of Nail Salon-Acquired *Mycobacterium fortuitum *Skin Infection. Clin Infect Dis.

[B2] Angeli K, Lacour JP, Mantoux F, Roujeau JC, Andre P, Truffot-Pernot C, Ortonne JP (2004). *Mycobacterium fortuitum *skin infection occurring after a facelift. Ann Dermatol Venereol.

[B3] Geertsma MF, Nibbering PH, Pos O, Van Furth R (1990). Interferon-gamma-activated human granulocytes kill ingested *Mycobacterium fortuitum *more efficiently than normal granulocytes. Eur J Immunol.

[B4] Nibbering PH, Pos O, Stevenhagen A, Van Furth R (1993). Interleukin-8 enhances nonoxidative intracellular killing of *Mycobacterium fortuitum *by human granulocytes. Infect Immun.

[B5] Christman MF, Morgan RW, Jacobson FS, Ames BN (1985). Positive control of a regulon for defenses against oxidative stress and some heat-shock proteins in *Salmonella typhimurium*. Cell.

[B6] Demple B, Halbrook J (1983). Inducible repair of oxidative DNA damage in *Escherichia coli*. Nature.

[B7] Gordon AH, Hart PD (1994). Stimulation or inhibition of the respiratory burst in cultured macrophages in a mycobacterium model: initial stimulation is followed by inhibition after phagocytosis. Infect Immun.

[B8] Garbe TR, Hibler NS, Deretic V (1996). Response of *Mycobacterium tuberculosis *to reactive oxygen and nitrogen intermediates. Mol Med.

[B9] Laochumroonvorapong P, Paul S, Manca C, Freedman VH, Kaplan G (1997). Mycobacterial growth and sensitivity to H_2_O_2 _killing in human monocytes in vitro. Infect Immun.

[B10] Zhang Y, Lathigra R, Garbe T, Catty D, Young D (1991). Genetic analysis of superoxide dismutase, the 23 kilodalton antigen of *Mycobacterium tuberculosis*. Mol Microbiol.

[B11] Cole ST, Brosch R, Parkhill J, Garnier T, Churcher C, Harris D, Gordon SV, Eiglmeier K, Gas S, Barry CE, Tekaia F, Badcock K, Basham D, Brown D, Chillingworth T, Connor R, Davies R, Devlin K, Feltwell T, Gentles S, Hamlin N, Holroyd S, Hornsby T, Jagels K, Krogh A, McLean J, Moule S, Murphy L, Oliver K, Osborne J, Quail MA, Rajandream M-A, Rogers J, Rutter S, Seeger K, Skelton J, Squares R, Squares S, Sulston JE, Taylor K, Whitehead S, Barrell BG (1998). Deciphering the biology of Mycobacterium tuberculosis from the complete genome sequence. Nature.

[B12] Dussurget O, Stewart G, Neyrolles O, Pescher P, Young D, Marchal G (2001). Role of *Mycobacterium tuberculosis *copper-zinc superoxide dismutase. Infect Immun.

[B13] Piddington DL, Fang FC, Laessig T, Cooper AM, Orme IM, Buchmeier NA (2001). Cu, Zn Superoxide Dismutase of *Mycobacterium tuberculosis *contributes to survival in activated macrophages that are generating an oxidative burst. Infect Immun.

[B14] Harth G, Horwitz MA (1999). Export of recombinant *Mycobacterium tuberculosis *superoxide dismutase is dependent upon both information in the protein and mycobacterial export machinery. J Biol Chem.

[B15] Edwards KM, Cynamon MH, Voladri RK, Hager CC, DeStefano MS, Tham KT, Lakey DL, Bochan MR, Kernodle DS (2001). Iron-cofactored superoxide dismutase inhibits host responses to *Mycobacterium tuberculosis*. Am J Respir Crit Care Med.

[B16] Volpe E, Cappelli G, Grassi M, Martino A, Serafino A, Colizzi V, Sanarico N, Mariani F (2006). Gene expression profiling on human macrophages at late time of infection with *Mycobacterium tuberculosis*. Immunology.

[B17] Menendez MC, Domenech P, Prieto J, Garcia MJ (1995). Cloning and expression of the *Mycobacterium fortuitum *superoxide dismutase gene. FEMS Microbiol Lett.

[B18] Miller RA, Britigan BE (1997). Role of oxidants in microbial pathophysiology. Clin Microbiol Rev.

[B19] Wayne LG, Diaz GA (1982). Serological, taxonomic and kinetic studies of the T and M classes of mycobacterial catalase. Intern J System Bacteriol.

[B20] Zhang Y (1996). Life without *kat*G. Trends Microbiol.

[B21] Slayden RA, Barry CE (2000). The genetics and biochemistry of isoniazid resistance in *Mycobacterium tuberculosis*. Microbes Infect.

[B22] Li Z, Kelley C, Collins F, Rouse D, Morris S (1998). Expression of *kat*G in *Mycobacterium tuberculosis *is associated with its growth and persistence in mice and guinea pigs. J Infect Dis.

[B23] Zhang Y, Heym B, Allen B, Young D, Cole S (1992). The catalase-peroxidase gene and isoniazid resistance of *Mycobacterium tuberculosis*. Nature.

[B24] Zhang Y, Garbe T, Young D (1993). Transformation with katG restores isoniazid-sensitivity in *Mycobacterium tuberculosis *isolates resistant to a range of drug concentrations. Mol Microbiol.

[B25] Smith I (2003). *Mycobacterium tuberculosis *pathogenesis and molecular determinants of virulence. Clin Microbiol Rev.

[B26] Dussurget O, Rodriguez M, Smith I (1998). protective role of the *Mycobacterium smegmatis Ide*R against reactive oxygen species and isoniazid toxicity. Tuber Lung Dis.

[B27] Zheng M, Doan B, Schnerider TD, Storz G (1999). OxyR and SoxRS regulation of *fur*. J Bacteriol.

[B28] Pagan-Ramos E, Song J, McFalone M, Mudd MH, Deretic V (1998). Oxidative stress response and characterization of the oxyR-ahpC and furA-katG loci in Mycobacterium marinum. J Bacteriol.

[B29] Zahrt TC, Song J, Siple J, Deretic V (2001). Mycobacterial FurA is a negative regulator of catalase-peroxidase gene *kat*G. Mol Microbiol.

[B30] Master S, Zahrt TC, Song J, Deretic V (2001). Mapping of *Mycobacterium tuberculosis kat*G promoters and their differential expression in infected macrophages. J Bacteriol.

[B31] Pym AS, Domenech P, Honore N, Song J, Deretic V, Cole ST (2001). Regulation of catalase-peroxidase (KatG) expression, isoniazid sensitivity and virulence by *fur*A of *Mycobacterium tuberculosis*. Mol Microbiol.

[B32] Milano A, Forti F, Sala C, Riccardi G, Ghisotti D (2001). Transcriptional regulation of *furA *and *katG *upon oxidative stress in *Mycobacterium smegmatis*. J Bacteriol.

[B33] Sala C, Forti F, Di Florio E, Canneva F, Milano A, Riccardi G, Ghisotti D (2003). *Mycobacterium tuberculosis *FurA autoregulates its own expression. J Bacteriol.

[B34] Menendez MC, Ainsa JA, Martin C, García MJ (1997). *katGI *and *katGII *encode two different catalases-peroxidases in *Mycobacterium fortuitum*. J Bacteriol.

[B35] Gibson UE, Heid CA, Williams PM (1996). A novel method for real time quantitative RT-PCR. Genome Res.

[B36] Heid CA, Stevens J, Livak KJ, Williams PM (1996). Real time quantitative PCR. Genome Res.

[B37] Johnson MR, Wang K, Smith JB, Heslin MJ, Diasio RB (2000). Quantitation of dihydropyrimidine dehydrogenase expression by real-time reverse transcription polymerase chain reaction. Anal Biochem.

[B38] Livak KJ, Schmittgen TD (2001). Analysis of relative gene expression data using real-time quantitative PCR and the 2(-ΔΔC^T^) Method. Methods.

[B39] Dubnau E, Fontan P, Manganelli R, Soares-Appel S, Smith I (2002). *Mycobacterium tuberculosis *genes induced during infection of human macrophages. Infect Immun.

[B40] Raynaud C, Papavinasasundaram KG, Speight RA, Springer B, Sander P, Bottger EC, Colston MJ, Draper P (2002). The functions of OmpATb, a pore-forming protein of *Mycobacterium tuberculosis*. Mol Microbiol.

[B41] Timm J, Post FA, Bekker LG, Walther GB, Wainwright HC, Manganelli R, Chan WT, Tsenova L, Gold B, Smith I, Kaplan G, McKinney (2003). Differential expression of iron-, carbon-, and oxygen-responsive mycobacterial genes in the lungs of chronically infected mice and tuberculosis patients. Proc Natl Acad Sci USA.

[B42] Gordillo S, Guirado E, Gil O, Diaz J, Amat I, Molinos S, Vilaplana C, Ausina V, Cardona P-J (2006). Usefulness of *acr *expression for monitoring latent *Mycobacterium tuberculosis *bacilli in *in vitro *and *in vivo *experimental models. Scand J Immunol.

[B43] Dubnau E, Chan J, Mohan VP, Smith I (2007). Responses of *Mycobacterium tuberculosis *to growth in mouse lung. Infect Immun.

[B44] Manganelli R, Dubnau J, Tyagi S, Kramer FR, Smith I (1999). Differential expression of 10 sigma factor genes in *Mycobacterium tuberculosis*. Mol Microbiol.

[B45] Menendez MC, Rebollo MJ, Nuñez MC, Cox RA, Garcia MJ (2005). Analysis of the precursor-rRNA fractions of rapidly growing mycobacteria: quantification by methods that include the use of a constitutive promoter (*rrn*A P1) as a novel standard. J Bacteriol.

[B46] Gomez M, Smith I, Hatfull GF, Jacobs WR Jr (2000). Determinants of Mycobacterial gene expression. Molecular genetics of Mycobacteria.

[B47] Smith I, Bishai WR, Nagaraja V, Cole ST et al (2005). Control of the mycobacterial transcription. Tuberculosis and the tubercle bacillus.

[B48] Gonzalez-y-Merchand JA, Garcia MJ, Gonzalez-Rico S, Colston MJ, Cox RA (1997). Strategies used by pathogenic and nonpathogenic mycobacteria to synthesize rRNA. J Bacteriol.

[B49] Gonzalez-y-Merchand JA, Colston MJ, Cox RA (1998). Roles of multiple promoters in transcription of ribosomal DNA: effects of growth conditions on precursor rRNA synthesis in micobacteria. J Bacteriol.

[B50] Schmittgen TD, Zakrajsek BA (2000). Effect of experimental treatment on housekeeping gene expression: validation by real-time, quantitative RT-PCR. J Biochem Biophys Methods.

[B51] Sala C, Forti F, Magnoni F, Ghisoti D (2008). The *kat*G mRNA of *Mycobacterium tuberculosis *and *Mycobacterium smegmatis *is processed at its 5' end and is stabilized by both a polypurine sequence and translation initiation. BMC Mol Biol.

[B52] Genomes OnLine Database. http://www.genomesonline.org.

[B53] Sherman DR, Sabo PJ, Hickey MJ, Arain TM, Mahairas GG, Yuan Y, Barry CE, Stover CK (1995). Disparate responses to oxidative stress in saprophytic and pathogenic mycobacteria. Proc Natl Acad Sci USA.

[B54] Calder KM, Horwitz MA (1998). Identification of iron-regulated proteins of Mycobacterium tuberculosis and cloning of tandem genes encoding a low iron-induced protein and a metal transporting ATPase with similarities to two-component metal transport systems. Microb Pathog.

[B55] Sambrook J, Russell DW (2001). Molecular cloning: A laboratory manual 3rd.

[B56] Wayne LG, Diaz GA (1986). A double staining method for differentiating between two classes of mycobacterial catalase in polyacrylamide electrophoresis gels. Anal Biochem.

[B57] Beauchamp C, Fridovich I (1971). Superoxide dismutase: improved assays and an assay applicable to acrylamide gels. Anal Biochem.

[B58] Wayne LG, Fridovich I (1987). Assaying for Superoxide dismutase activity: some large consequences of minor changes in conditions. Anal Biochem.

[B59] Gonzalez-y-Merchand JA, Colston MJ, Cox RA (1996). The rRNA operons of *Mycobacterium smegmatis *and *Mycobacterium tuberculosis*: comparison of promoter elements and of neighbouring upstream genes. Microbiology.

[B60] Movahedzadeh F, Gonzalez-y-Merchand JA, Cox RA, Parish T, Stoker NG (2001). Transcription start-site mapping. Mycobacterium tuberculosis protocols.

[B61] Mironov VN, Van Montagu M, Inzé D (1995). High throughput RNase protection assay. Nucleic Acids Res.

[B62] Winer J, Jung CK, Shackel I, Williams PM (1999). Development and validation of real-time quantitative reverse transcriptase-polymerase chain reaction for monitoring gene expression in cardiac myocytes in vitro. Anal Biochem.

