# Supplementary material for: Transcriptional analysis of Mycobacterium fortuitum cultures upon hydrogen peroxide treatment using the novel standard rrnA-P1
Source: BMC Microbiol. 2008 Jun 19;8:100. doi: 10.1186/1471-2180-8-100 (PMC2443159; doi:10.1186/1471-2180-8-100)
Supplement: Additional file 1 — Quantitative data corresponding to Figures 1 and 3. Data corresponding to Figure 1 show the densitometry of a representative activity gel of KatG and SOD in protein extracts of Mycobacterium fortuitum. Data corresponding to Figure 3 show the cpm values of a representative experiment of hybridization of a Northern Blot with KatGII, sodA and rrs (16SrRNA) before and after normalization. [file 1471-2180-8-100-S1.pdf]

NUÑEZ et al. "Transcriptional analysis of *Mycobacterium fortuitum* cultures upon hydrogen peroxide treatment using the novel standard *rrn*A-P1". 2008.

ADDITIONAL FILE 1

DATA corresponding to Figure 1.
Densitometry of a representative gel of proteins' activity.

	**KatGI**	**KatGII**	**SodA**
0.02mM 30min	19.12	85.4	109.2
0.02mM 60min	22.5	80.7	103.9
0.02mM 120min	19.66	89.34	110.9
2mM 30min	16.4	62.3	102.6
2mM 60min	16	71.02	123.8
2mM 120min	14.9	76.21	113.8
20mM 30min	20.67	30.8	113.8
20mM 60min	20.8	42.8	128.6
20mM 120min	21.6	68.1	124.4
Control	100	100	100

DATA corresponding to Figure 3.
Radioactive cpm values obtained in a representative experiment using Northern Blot analysis.

Cpm values before normalization

	***rrs* (16S)**	**katGII**	**sodA**
0.02mM 30min	36	18.93	45
0.02mM 60min	40	22.4	34
0.02mM 120min	66	20.8	67
0.2mM 30min	54	48.16	68
0.2mM 60min	60	32.48	63
0.2mM 120min	56	28	68
2mM 30min	72	41.36	70
2mM 60min	72	30.4	71
2mM 120min	72	66.4	78
20mM 30min	70	320	113
20mM 60min	62	45.6	63
20mM 120min	66	33.6	102
Control	70	34.4	62

Cpm values after normalization with *rrs*(16S)

	***kat*GII**	***sod*A**
0.02mM 30min	0.53	1.25
0.02mM 60min	0.56	0.85
0.02mM 120min	0.32	1.01
0.2mM 30min	0.89	1.26
0.2mM 60min	0.54	1.05
0.2mM 120min	0.5	1.21
2mM 30min	0.57	0.97
2mM 60min	0.42	0.89
2mM 120min	0.92	1.08
20mM 30min	4.57	1.61
20mM 60min	0.73	1.01
20mM 120min	0.51	1.54
Control	0.49	0.88
